# A New Approach for the Diagnosis of Systemic and Oral Diseases Based on Salivary Biomolecules

**DOI:** 10.1155/2019/8761860

**Published:** 2019-02-17

**Authors:** Alexandra Roi, Laura C. Rusu, Ciprian I. Roi, Ruxandra E. Luca, Simina Boia, Roxana I. Munteanu

**Affiliations:** ^1^Department of Oral Pathology, “Victor Babes” University of Medicine and Pharmacy, Timisoara, 2 Eftimie Murgu Sq., 300041, Romania; ^2^Department of Anaesthesiology and Oral Surgery, “Victor Babeș” University of Medicine and Pharmacy, Timisoara, 2 Eftimie Murgu Sq., 300041, Romania; ^3^Department of Oral Rehabilitation and Dental Emergencies, “Victor Babeș” University of Medicine and Pharmacy, Timisoara, 2 Eftimie Murgu Sq., 300041, Romania; ^4^Department of Periodontology, “Victor Babeș” University of Medicine and Pharmacy, Timisoara, 2 Eftimie Murgu Sq., 300041, Romania

## Abstract

Early diagnosis represents the target of contemporary medicine and has an important role in the prognosis and further treatment. Saliva is a biofluid that generated a high interest among researchers due to its multiple advantages over other body fluids. The multitude of components that can act as biomarkers influenced the existing technologies to develop protocols that could allow saliva to become the new noninvasive diagnostic method. Saliva as a diagnostic tool can bring substantial addition to the diagnostic armamentarium, providing important information about oral and general health. The diagnostic applications of saliva extended and had a rapid evolution due to the advancement in salivaomics. The present review summarizes the latest researches in saliva-related studies and explores the information and correlations that saliva can offer regarding the systemic and oral diseases, highlighting its great potential of diagnosis. It is expected that in the future specific guidelines and results regarding the salivary diagnostics are to be available, together with high-sensitivity and specificity tests for multiple systemic and oral diseases.

## 1. Introduction

Body fluids provide a wide perspective regarding the biological processes and the health of different organs. The human body is composed of a variety of fluids, such as blood, urine, and saliva, with a high quantity of proteins that can be associated with several systemic and oral diseases. These fluids proved to have found widespread clinical applications in order to diagnose and monitor human health. The high global impact of a large number of diseases including cancer and cardiovascular, metabolic, and neurological diseases challenged the clinicians to provide and improve the diagnosis procedures and clinical evaluation of these patients. One of the most appealing diagnostic tools is thought to be the human saliva, holding the key to an early diagnosis, a better treatment, and an improved prognosis [1]. The early detection of the diseases is often a difficult task and implies more clinical and laboratory investigations that can delay the treatment and highly influence the prognosis.

Systemic diseases are very challenging to diagnose without more invasive supplementary investigations. In order to overcome this condition, medical researchers worked into finding molecular disease biomarkers that can be easily identified and where they can successfully implement a noninvasive and fast diagnosis. During this path of research, three main limitations have influenced until recent the late development and research of specific biomarkers for early disease detection: (1) the lack of definitive molecular biomarkers for specific diseases, (2) the lack of an easy and inexpensive sampling method with minimal discomfort, and (3) the lack of an accurate and easy-to-use platform that can facilitate the early detection. Until now, it can be considered that limitations 1 and 3 have found solutions with the help of salivary biomarkers and an ongoing development of salivary diagnosis [2].

Salivary diagnosis is viewed as a promising modality that can provide an early and accurate diagnosis, an improved prognosis, and a good monitoring post-therapy. The whole saliva is composed of the secretions of the minor and major salivary glands as well as mucosal transudations, gingival crevicular fluid, serum and some blood derivatives, desquamated epithelial cells, bacteria, viruses, fungi, and food debris. Saliva is a complex fluid that also contains a high number of hormones, proteins, enzymes, antibodies, cytokines, and antimicrobial constituents that can facilitate their associations with a variety of systemic diseases [1]. The assay of saliva represents a wide area of research at this time and has implications that target basic and clinical purposes. The indications suggest that saliva can be used as an investigative tool for disease processes and disorders, and after a careful analysis, it can provide multiple information about the functioning of the organs within the human body [3].

The past research within the last 10 years proves the fact that saliva as a diagnostic tool has gained a lot of attention and has become a translational research method. Saliva has the potential to become a first-line diagnostic tool with the help of the advancement made in early detection and the development of biomolecules that have clinical importance [4]. Salivary diagnostics has received attention due to its connections to various high-impact systemic diseases and physiological conditions that were shown to have an influence in the composition of saliva. Serious investments were made, motivating scientists, governments, and industry to direct resources in the saliva diagnostics [2]. A good method for salivary diagnostics should have general functionality, high sensitivity and specificity, low cost, and efficient clinical application. Regarding saliva, many of these requirements have been accomplished with the implication of several fields such as chemistry, physics, biology, and engineering, in order to develop an accurate and efficient test [2].

Saliva has several advantages over serum and tissue fragments in its use as a diagnostic tool. One of the most appealing characteristics is the noninvasive approach that, combined with the easy collection method and storage, makes it a valuable tool. New technologies have proven their efficacy and unveiled a large number of salivary biomarkers that are connected to several general and oral diseases [5].

The aim of this review is to emphasize the role and importance of saliva as a diagnostic tool for the diagnosis of systemic and oral diseases. The use of this method brings to light an efficient and easy approach that can improve considerably the diagnosis, prognosis, treatment, and post-therapy monitoring. Various components in this fluid can act as biomarkers for multiple diseases providing valuable information regarding the health status. The focus is on providing information about the important salivary constituents, the mechanism of using saliva as a diagnostic tool, and the clinical applications that can influence an early diagnosis.

## 2. Biomarkers Era: An Evolution

The definition of a biomarker refers to a pharmacological or physiological measurement that can be used to predict a toxic event, in this specific case a molecule that contains particular material that can be used in order to diagnose a disease or measure the progression and treatment outcome. The characteristics of biomarkers make them proper for an alternative diagnostic tool, with or without the help of other methods [6].

The development of mass spectrometric technologies led medicine to a new era in biomarker discovery that will have an important impact on future disease diagnosis and therapy. More studies in salivary proteins showed the fact that saliva contains actually hundreds of minor proteins or peptides that although are present in variable concentrations can have a significant role in the diagnosis of diseases; these proteins can receive the role of biomarkers in relation to specific conditions. Although proteomes play an important role in the diagnosis, the salivary transcriptomic technology succeeded to improve the diagnostic potential of saliva for multiple medical applications [2].

Proteomic technology helped to discover the salivary biomarkers by outlining the importance of the proteome and the analysis of the expressed proteomics. The existence of the proteomes in the body fluids represents a high potential of disease markers. An accurate analysis of the human saliva proteome can be related to the general health status. Many functional alterations of proteins result from posttranslational modifications such as phosphorylation, glycosylation, acetylation, and methylation [2]. These kinds of alterations and modified proteins can be specific in some diseases such as autism spectrum disorder [7] and cervical cancer [8].

The transcriptomic technology allowed researchers to discover the salivary transcriptomes (RNA molecules) that include the molecules the cells use to transport information provided by the DNA for protein production. This opportunity provides medical research with a second diagnostic tool that involves saliva and that can provide more opportunities for salivary diagnostics [2].

## 3. Salivary Biomarkers: Generalities

The most important and revealing components of the saliva are the proteins. Human saliva has a specific proteomic content that allows researchers to perform assays in order to discover novel saliva biomolecules associated with general health status. Proteomic studies of saliva help with the identification of new proteins and peptides that can help quantify the biological activity in pathological states.

The Saliva Proteome Knowledge Base (http://www.skb.ucla.edu) is the first database that contains all the proteomic data being accessible to the public. The techniques used by researches and biochemists in order to perform the proteome work from saliva are gel electrophoresis, capillary electrophoresis, nuclear magnetic resonance, MS, immunoassay, and LC [9]. Due to the great development, researchers have proposed the term salivaomics. This specific term gathers all the technologies used for analyzing potentially salivary biomarkers: proteomics, genomics, transcriptomics, microRNA (miRNA), and metabonomics [10]. The value of salivary biomarkers has long been overcome until recent research on upgraded saliva from the position of being useless to the one of being a high-sensitivity diagnostic method. Research proved the high potential of the salivary biomarkers and their diagnostic capability, promoting it with uncontestable advantages over other body fluids.

## 4. Particularities of Saliva: Composition, Functions, and Production

Saliva is a unique fluid that contributed to the development of a new diagnostic tool in the past few years. The research has shown that a wide spectrum of hormones, nucleic acids, electrolytes, and proteins/peptides can be related to multiple local and systemic diseases. It is said that saliva reflects the “body's health” and well-being, but until recently its use as a diagnostic tool has been hindered because the examination of the biomolecules that exist in saliva and their relevance and association with different etiologies has been not enough explored [4]. Used for the diagnosis of systemic diseases, saliva is an important advantage, primarily because saliva contains a small amount of plasma. Plasma-derived biomarkers in saliva facilitate the continuous monitoring of the oral and general health status [11].

The salivary fluid is an exocrine secretion that consists of approximately 99% water, with a variety of electrolytes (sodium, potassium, calcium, magnesium, and phosphate), proteins such as enzymes, immunoglobulins, antimicrobial factors, albumin, polypeptides and oligopeptides, traces of albumin, and mucosal glycoproteins of great importance in maintaining a balance of the oral health. Saliva also contains glucose, urea, and ammonia in various quantities that can interact and be responsible for several general diseases [12].

The oral fluid originates preponderantly from three pairs of major salivary glands (parotid, sublingual, and submandibular) and from numerous minor salivary glands. Parotid glands are serous glands, and their secretion lacks mucin; the submandibular and sublingual glands are mixed ones, with ser-mucous secretion. Minor salivary glands that are situated in the connective tissue below the circumvallate papillae are Von Ebner glands, and the mucous ones are Blandin-Nühm glands [13].

The salivary composition varies and depends on the type of the gland, mucous or serous ones [14]. Its composition differs by the contribution of each gland in order to obtain the total of unstimulated saliva secretion, and the variations are from 65%, 23%, and 8% to 4% for the submandibular, parotid, Von Ebner, and sublingual glands [3]. Components of saliva can have also a nonglandular origin; basically, the oral fluid is considered to be a mixture of the production of salivary glands and other fluids that originate from the oropharingeal mucosa (oral mucosal transudate, fungi, bacteria, viruses, and gastrointestinal reflux liquid) [15, 16]. To the total composition, there is also a contribution from the crevicular fluid (a fluid that derivates from the epitheliul of the gingival crevice) that is produced at approximately 2-3 *μ*l/h per tooth and it can be considered as a plasma transudate. The oral fluid also can contain food debris and blood-derivated compounds such as plasmatic proteins, erythrocytes, and leucocytes in case there is inflammation present [3]. The composition of saliva based on its constituents is inorganic, organic nonprotein, protein/polypeptide, hormone, and lipid molecules [17, 18] (Table 1).

The number of total protein increases in the salivary secretion through *β*-sympathetic activity in the salivary glands, since saliva secretion is mainly evoked by the action of adrenergic mediators [19]. Saliva contains a large number of protein compounds, and their structure and function have been studied with biochemical techniques, including liquid chromatography, gel electrophoresis, capillary electrophoresis (CE), nuclear magnetic resonance, mass spectrometry, immunoassays (RIA, IRMA, EIA, and ELISA) and lectin probe analysis [10, 20] (Table 2). Along with time, with the help of proteomic techniques, complete patterns of all the salivary proteins were accomplished.

Researchers that focused on the study of human saliva have characterized 4 major types of salivary proteins: PRPs, cystatins, statherins, and histatins. The important role of this type of proteins is maintaining the integrity of tooth structures in the oral cavity, especially involved in the demineralization and remineralization process of the enamel [4].

In the oral fluid, hormones that are especially detected in plasma can also be present. Although certain correlations have been made, further studies are necessary in order to prove the connection of salivary hormone level with the plasma ones so it can be a trustful association with pathological and physiological states. At the present time, there is still few information regarding the association of salivary hormones and different pathologies, but until now steroid detection is a promising application in salivary hormonal studies. The most commonly assayed salivary biomarkers are cortisol, testosterone, progesterone, aldosterone, and hydroxyprogesterone [3]. Salivary cortisol measurement is nowadays an accepted alternative, proved by the fact that both serum and salivary levels are equivalent. There were also important advancements made, proving the existence of growth hormone, prolactine, and insulin-like growth factor I with similar levels to those found in serum directing the research to exploiting new fields of interest [3].

## 5. Saliva as a Diagnostic Tool: Introduction into a New Perspective

The use of saliva as a diagnostic fluid has gained attention in the past few years, and researches have proved the high sensitivity of this type of diagnosis regarding the detection and prediction of diseases. As a diagnostic fluid, saliva offers several advantages over serum: being a cost-effective approach, having real-time diagnostic values, having multiple samples which can be obtained easily, requiring less manipulation during the diagnostic procedure, and having a noninvasive collection method with a minimal risk of infections and addressing to all categories of patients, especially those to whom blood sampling could be a challenge (children, anxious or uncooperative patients) [4]. In this review, we would like to outline the diagnostic potential of saliva and its implication in the detection of several diseases taking into consideration the high-quality DNA that this fluid possesses.

Saliva is an important fluid, and interest in it has developed due to the wide spectrum of proteins/peptides, electrolytes, hormones, and nucleic acids that are in its composition and can provide important information about the body's health. The delay in the use of saliva as a diagnostic method was mainly because until recent there has been a lack of understanding of the biomolecules that were found in the saliva. As a diagnostic tool, several disadvantages have been reported: the variations due to the diurnal/circadian rhythm, the method of collection that can influence the salivary composition, and the necessity of sensitive detection systems. However, saliva is considered to have an enormous potential of biomarkers that range from changes in biochemical, DNA, RNA, and proteins to the oral environment. As a diagnostic tool, saliva can provide a new and noninvasive perspective in order to obtain a diagnosis, and it can be expected in the future to become a substitute for serum and urine tests [21]. A part of the constituents enter the saliva through blood by passive/active transport or extracellular ultrafiltration [22].

Clinical research has developed various protocols in order to assay saliva. At the time, saliva is most frequently used as a diagnostic tool for systemic diseases and the future relies in combinations of different biomarker panels that can be used for screening in order to improve the early diagnosis and the general outcome [4]. The first choices in the analysis are the proteomic constituents, but genomic targets can be a valuable source of biomarkers also. Salivary diagnostics with the help of biotechnologies made it possible for several biomarkers to be associated with multiple diseases such as cancer, autoimmune diseases, viral diseases, bacterial diseases, cardiovascular diseases, and HIV. The clinical need of a simple and easy diagnostic tool is sadly lacking although we are surrounded by multiple health risks and diseases. Saliva used as a diagnostic is an important challenge based on the need to identify diagnostic markers that can be successfully used in a clinic.

## 6. Potentially Salivary Biomarkers for Oral and Systemic Diseases

For many years now, researchers investigated the importance of the changes that occurred in the saliva, changes that affect the flow rate and composition. The changes in the fluid are valuable regarding the diagnosis of oral and systemic diseases [23]. At first, the examination of saliva was used in order to identify the local gland diseases, such as inflammatory and autoimmune diseases [24], but later on the researchers expanded their work, highlighting the potential for diagnosing multiple general diseases.

### 6.1. Periodontal Disease

Regarding the periodontal pathogenic processes, periodontitis can be classified based on the three phases of evolution: inflammation, connective tissue degradation, and bone turnover. There are, associated with each phase of the periodontal disease, different salivary biomarkers that can stage the evolution and the status of the patient. At the beginning of the inflammatory phase, prostaglandin E2, interleukin-1, interleukin-6, and tumor necrosis factor-alpha are found in a high number, released from a variety of cells [25]. As the stages progress and the disease becomes more advanced with severe bone loss, the levels of tumor necrosis factor, interleukin-1, and RANKL are elevated and directly related to the degree of bone destruction [25]. The specific biomarkers for the bone, such as pyridinoline cross-linked carboxyterminal telopeptide of type I collagen, are being transported in the crevicular fluid into the periodontal pocket and finally become a component of saliva [26, 27].

An important cytokine with a proinflammatory role involved in the inflammation process associated with periodontitis is interleukin-1. IL-1 can be the product of several cells, as epithelial cells, monocytes, polymorphonuclear neutrophils, fibroblasts, endothelial cells, and osteoblasts [28, 29]. Interleukin-1 influences the production of prostaglandin E2 and is involved in the regulation of metalloproteinases and their inhibitors, and it induces the osteoclastic activity that sustains bone loss associated with periodontitis [28, 30, 31]. The entire activity of IL-1 is based on interleukin-1alpha and interleukin-1beta (was proved to be elevated in association with periodontitis) [31–33]. Also, studies found increased salivary levels of IL-6 in patients diagnosed with periodontitis [34–36] and proved the fact that it influences osteoclast differentiation and bone resorption, being directly involved in tissue destruction [37, 38].

Another key biomarker involved in periodontitis is mainly produced by macrophages and is represented by tumor necrosis factor-alpha. It is an important immune mediator, and in relationship with this disease, it influences bone collagen synthesis and induces collagenases, similar to IL-1 [28, 39]. Also involved in the periodontal disease, matrix metalloproteinase-9 is part of the process of periodontal disease, especially immune response and tissue degradation [40–42]. The elevated salivary levels of matrix metalloproteinase-9 prove that the characteristics of a biomarker are being accomplished and associated with disease conditions, as low salivary levels are associated with a clinically normal condition [40, 43] (Table 3).

A recent study outlined the existence of certain correlations between salivary superoxide dismutase levels and the gingival index, pocket depth, and clinical attachment loss found in patients that were diagnosed with chronic periodontitis. Saliva's potential of diagnosis is seen as a noninvasive and easy way to diagnose patients with premalignant conditions [44]. Also, salivary macrophage inflammatory protein-1*α*, matrix metalloproteinase-8, interleukin- (IL-) 1*β*, IL-6, prostaglandin E2, and tumor necrosis factor- (TNF-) *α* levels seem to be associated with gingivitis and periodontitis, having a high potential to be used in their diagnosis [45]. Based on another study, the salivary levels of uric acid, transaminase, procalcitonin, IL-8, and Toll-like receptor-4 were higher in patients diagnosed with periodontitis than in the healthy control group, proving positive correlations between the gingival index, pocket depth measurements, and clinical attachment loss (Table 4) [46, 47]. More recently, a new oral rinse system has been developed that can effectively estimate the number of neutrophils found in the saliva in order to certify the existence of periodontal disease [48].

### 6.2. Sjögren's Syndrome

Sjögren's syndrome (SS) is an autoimmune chronic systemic disease that has important symptoms: xerostomia and keratoconjunctivitis. Patients diagnosed with SS have a decreased salivary flow rate and a modified composition of the saliva. It was shown the fact that this syndrome is accompanied with significant changes in the proteome and transcriptome, having also important alterations in the levels of IL-4, IL-5, and cytokine clusters [32]. Another important research identified 19 genes (EPSTI1, IFI44, IFI44L, IFIT1, IFIT2, IFIT3, MX1, OAS1, SAMD9L, PSMB9, STAT1, HERC5, EV12B, CD53, SELL, HLA-DQA1, PTPRC, B2M, and TAP2) that were correlated with this syndrome and were responsible for the induction of interferons and antigen presentation [49]. The study of Hu et al. identified a panel of biomarkers that had high levels in patients with SS, including a number of three mRNA biomarkers (guanylate-binding protein 2, myeloid cell nuclear differentiation antigen, and low-affinity IIIb receptor for the Fc fragment of IgG) [50]. These types of biomarkers from the transcriptome and proteome can provide in the future a simple diagnostic tool for SS.

### 6.3. Oral Cancer

Early diagnosis and treatment is the key to a good prognosis in almost all types of cancer. Saliva has been used in studies as a diagnostic tool for oral squamous cell carcinoma (OSCC) based on the help of salivary analytes (proteins, mRNA, and DNA) [1]. Oral cancer is the sixth most common cancer type worldwide, and 90% is represented by OSCC. The average 5-year survival rate is approximately 60% [51], and usually the high mortality rate is associated with a late diagnosis. The solution for the future is to develop strategies to obtain an early diagnosis for OSCC. Until now, several biomarkers have been reported in association with OSCC, including IL-8, endothelin receptor type B hypermethylation [52], and microRNAs (such as miR-200a, miR-125a, and miR-31) [53–55]. Other previous salivary transcriptomic studies have discovered seven oral squamous cell carcinoma-associated salivary RNAs (S100 calcium-binding protein P, dual-specificity phosphatase 1, interleukin-8, interleukin-1beta, H3 histone family 3A, ornithine decarboxylase antizyme 1, and spermine N1-acetyltransferase) that showed a prediction accuracy of 81% as biomarkers for OSCC [56]. More research studies proved the importance of three tumor markers (Cyfra 21-1, tissue polypeptide antigen (TPA), and cancer antigen CA125) that were found to have a high level in the saliva of patients diagnosed with OSCC [57].

The existence of gene mutations can often be associated and used as biomarkers in order to diagnose oral cancer. In saliva, the tumor-specific DNA was positive in 100% of the patients diagnosed with oral cancer, and 47-70% of the patients with tumors in other places of the body also carry specific tumor DNA markers in the saliva [21].

The *p53* protein is responsible for tumor suppression, and it is produced in cells as a response to multiple DNA damages. The inactivation of p53 during a mutation is one of the main causes of the development of malignancy. Studies have shown the fact that p53 antibodies were detected in the saliva of patients diagnosed with oral squamous cell carcinoma [58]. CA 125 is a tumor-associated antigen that was found in high levels in the saliva of the patients with oral, breast, and ovarian cancer [59]. Also, an important aspect is the fact that salivary cortisol levels were found to be significantly high in the saliva of patients diagnosed with OSCC. This association suggests that this hormone can be used as a marker for clinical staging [60].

It can be affirmed the fact that all the results prove that saliva has an important charge of biomarkers that can be used successfully in providing a screening and diagnosis of oral cancer.

### 6.4. Cardiovascular Disease

Cardiovascular disease (CVD) includes atherosclerosis, coronary heart disease, and myocardial infarction. The studies performed by Kosaka et al. [61] show that the salivary levels of IL-1*β*, IL-6, TNF-*α*, and prostaglandin E2 are increased in atherosclerosis, suggesting that these cytokines could be potential biomarkers in the diagnosis of atherosclerosis. Other studies concluded the fact that other salivary markers can be C-reactive protein (CRP), myoglobin (MYO), creatine kinase myocardial band (CKMD), cardiac troponins (cTn), and myeloperoxidase. Acute myocardial infarction was predicted by a correlation of an ECG with the CRP levels, proving 80% sensitivity and 100% specificity [62]. In saliva, there were also CK-MB and troponins identified, but their diagnostic potential was very low [63]. Also, the levels of *α*-2-HS-glycoprotein in saliva seem to decrease in patients diagnosed with cardiovascular diseases, suggesting the fact that the peptidome can contribute to the early diagnosis of these patients [64].

### 6.5. Alzheimer and Other Neurodegenerative Disorders

Alzheimer's disease (AD) is one of the most common neurovegetative disorders that occur to the aging population. It is supposed that the process of Alzheimer's is initiated years before it becomes clinically manifest [65]. Until now, the specific biomarkers for this disease could be found in the cerebrospinal fluid through the amyloid b levels [66] or using structural and functional magnetic resonance imaging [67], procedures that proved to be invasive and time-consuming. Further researches show that the existence of Ab and tau [68, 69] or a-Syn and DJ-1 [70] in human saliva can be considered proteins that are related to Alzheimer's disease and Parkinson's disease, suggesting actually the implication of saliva and its potential in the diagnosis of neurodegenerative diseases. Risk factors in the development of Alzheimer's disease are systemic infections [71], brain infections due to bacteria or virus involvement [72], but the association of various antimicrobial peptides in this disease is still not completely clear.

The study performed by Carro et al. [73] investigates the potential of lactoferrin as a salivary biomarker for Alzheimer's, based on the fact that lactoferrin is an antimicrobial peptide that targets bacteria, fungi, protozoa, viruses, and yeasts [73–75]. The results of their study show that lactoferrin can be used as a biomarker for Alzheimer's disease, after the outcome was compared to a standard test performed for the certain diagnosis of AD, proving a very high correlation with validated cerebrospinal fluid biomarkers. Although more studies are needed, lactoferrin has proved its correlations and has the potential of being a solid biomarker that can help the screening process of “apparently healthy” individuals that can suffer from a preclinical stage of the disease [73].

Ahmadi-Motamayel et al. [76] conducted a recent study with the aim at evaluating acetylcholinesterase (AChE) and pseudocholinesterase (PChE) in whole saliva in patients with Alzheimer's disease and in healthy subjects. Until now, many studies have been performed focusing on the salivary biomarkers in Alzheimer's disease and only a few regarding the salivary cholinesterase enzyme. The result of this study after the comparison of the salivary samples of the healthy subjects and those diagnosed with Alzheimer's disease concluded the fact that AChE and PChE levels were increased in saliva samples of patients with Alzheimer's disease [76].

Parkinson's disease is characterized pathologically by progressive degeneration of dopaminergic (DA) neurons in the substantia nigra pars compacta. The formation of a-synuclein- and ubiquitin-containing fibrillar inclusions (Lewy bodies and Lewy neurites) occurs in this cell population as well as variable changes in other neurotransmitter systems [77]. The aim of the research initiated by Song et al. [77] was to evaluate the levels and implications of salivary HO-1 in patients with idiopathic Parkinson's disease. The results showed that salivary HO-1 concentrations are significantly elevated in patients with idiopathic PD versus nonneurological controls matched for sex. Importantly, the test most effectively differentiated controls from Parkinson's disease patients at the earliest motor stages of the disease and was not influenced by age, sex, and various comorbidities.

### 6.6. Viral Infections

The existing tests for viral infections are based on salivary biomarkers, basically on viral DNA and RNA, antigens, and antibodies. Currently, several salivary tests are available based on the proteomic analysis of the saliva and the existing antibodies for hepatitis A, B, C viruses, HIV-1, rubella virus, mumps virus, and others [21]. A new salivary test is used by the san Raffaele Scientific Institute in Milan that is named OraQuick hepatitis C virus and represents a fast antibody test in order to detect easily the presence of the virus [78]. Nefzi et al. [79] conducted a study that showed the fact that human cytomegalovirus (HHV-6) appears to be more easily identified in saliva than in serum.

The HIV (human immunodeficiency virus) is possible through antibody-based screening assays. The diagnostic test is an antibody assay that can be a Western blot test via blood or saliva or a polymerase chain reaction via blood [1]. These specific tests have the aim at identifying the p24 antigens and antibodies against HIV-1 and HIV-2. However, the detection of viral RNA is difficult to be performed during salivary analysis due to the decreased viral load [80].

### 6.7. Lung Cancer

Early diagnosis is an important aspect regarding this type of cancer knowing the fact it is the most common cause of death in men and women. Until now, conventional diagnosis methods are not suited for screening, with a high false-negative rate [81, 82]. CT is being used for routine screening for early lung cancer, with the disadvantage of a high false-positive rate [83]. Salivary biomarkers have the potential to help the early diagnosis without using CT [84]. After studies were performed, 16 potentially biomarkers have been discovered that can efficiently contribute to the salivary diagnosis [85], three of them (haptoglobin, calprotectin, and zinc-a-2-glycoprotein) with a high sensitivity and excellent specificity. The transcriptomic biomarker profile—B-Raf gene, cyclin I, the EGF receptor, FGF-19, fibroblast growth factor receptor substrate 2, growth regulation by estrogen in breast cancer 1, and leucine zipper putative tumor suppressor 1—has been identified, and a panel of five of these biomarkers accomplished to achieve a sensitivity of 93.75% and a specificity of 82.81% regarding the diagnosis of lung cancer [86].

### 6.8. Orofacial Pain Salivary Biomarkers

The tissues that are found in the orofacial region are heterogeneous, a fact that makes the treatment of pain conditions a challenge for the clinician. The main problem for an adequate treatment option consists in the diversity of conditions for which orofacial pain is a major symptom that makes it hard to differentiate many of these disorders clinically [87]. Several population-based cross-sectional studies revealed a 1-month prevalence rate of self-reported orofacial pain that varies from 19% to 26% [88, 89]. The current research must focus on methods that combine different biomarkers for a condition. Biomarkers evaluation combines physiological parameters, psychological and behavioral aspects, genomics, and molecular and protein characteristics [87].

Orofacial pain is a sensory experience within a specific anatomical region and can be related to some common chronic orofacial entities: TMJ myalgia and arthralgia, atypical odontalgia, persistent dentoalveolar pain disorder, burning mouth syndrome, persistent idiopathic facial pain, neuralgia of the head and neck, and primary headache syndromes [87].

Collecting saliva in order to identify biomarkers associated with orofacial pain is a painless method and is easy to collect and store. Recently, saliva and synovial fluids have piqued the interest of numerous researchers and clinicians as possible alternatives to serum.

Further researches conclude that saliva-based biomarkers are not only preferred but also are accurate in discerning healthy subjects from those afflicted with periodontal disease or burning mouth syndrome [90–94]. Saliva has also been used as an indicator of stress and chronic pain. Several studies report substance P, a neuropeptide associated with inflammation status and pain, as well as the stress hormone cortisol, and markers of oxidative stress can be repeatedly detected within salivary secretions [95, 96].

The study performed by Jasim et al. focused on salivary biomarkers related to chronic pain by comparing blood samples and saliva samples of the same subjects and revealed the fact that five specific biomarkers related to pain were targeted. The results showed that they were first to find several isoforms of NGF, CGRP, and BDNF in saliva. The expression showed great variations between different saliva collection methods [97]. Glutamate was mostly expressed in whole stimulated saliva, and in contrast, the concentration was moderately correlated between saliva types as well as in plasma. The concentration of glutamate has also been shown to be elevated in different pain conditions [98, 99]. These results suggest that glutamate may be an essential pain mediator in peripheral tissue and may therefore act as a potential pain biomarker among others.

With the help of a standardized collection procedure and protocol, the use of salivary biomarkers for different orofacial pain disorders is a promising diagnostic method that will allow for a noninvasive approach.

## 7. Conclusions

Saliva is an important biological fluid with a wide area of research and applications, having a high potential to become the future in early diagnosis. The effective contribution of genomic and proteomic technologies made possible for saliva to become an attractive solution to other invasive diagnostic methods. Saliva as a diagnostic tool for oral and systemic diseases has multiple advantages over other body fluids and based on specific biomarkers can provide an accurate diagnosis. However, until saliva becomes a certified diagnostic test that can replace the conventional ones, all the research values must be compared with the existing accepted methods. The main problem consists in the fact that a standardized and accurate method of saliva collection needs to be associated with each type of diagnostic test, in order to avoid errors. This review has discussed several oral and systemic diseases that could be diagnosed based on different salivary biomarkers, but research needs to be extended in order for saliva to become an effective and sure diagnostic tool that can be used for screening and uncontestable diagnosis.

## Figures and Tables

**Table 1:** Comparison of inorganic compounds between saliva and plasma [3].

Inorganic compounds (mmol/l)	Whole unstimulated saliva	Whole stimulated saliva	Plasma
Na+	5	20- 80	145
K+	22	20	4
Cl−	15	30–100	120
Ca^2+^	1–4	1–4	2.2
HCO_3 −_	5	15–80	25
Mg^2+^	0.2	0.2	1.2
NH_3_	6	3	0.05

**Table 2:** Salivary proteins [3].

	Origin	Functions	Concentrations
Total proteins			0.47 ± 0.19 mg/ml
			0.9 ± 0.2 mg/ml
			4.3–710.0 mg/dl
			2.67 ± 0.54 mg/ml

*α*-Amylase		Starch digestion	3257 ± 1682 U/ml
			1080.0 ± 135.6 IU/I
			476 ± 191 *μ*g/ml

Albumin	Plasma	Mainly from plasma leakage	0.2 ± 0.1 mg/ml
			0.8–192.0 mg/dl

Cystatin group	SM>SL	Antimicrobial (cistein-proteinase inhibitor)	14.3 kDa form
			58 ± 25 *μ*g/ml
			14.2 kDa form
			91 ± 46 *μ*g/ml

Hystatin	P	Antifungal	1190 ± 313 *μ*g/ml

Secretory-IgA	B lymphocytes	Antimicrobial	124.3–335.3 *μ*g/ml

Lactoferrin	Mucous>serous	Antimicrobial	3.7 ± 2.5 *μ*g/ml

Lysozyme	SL>SM, P	Antimicrobial	3.5–92.0 *μ*g/ml
			21.8 ± 2.5 mg/dl
			59.7–1062.3 *μ*g/ml

PRPs	P	Binding to bacteria and with dietary tannins	Acidic PRP: 456 ± 139 *μ*g/ml
			Basic PRP: 165 ± 69 *μ*g/ml

Statherin		Ca++ binding	4.93 ± 0.61 *μ*mol/I
			36 ± 18 *μ*g/ml

Transferrin	Plasma		0.58 ± 0.20 mg/dl

**Table 3:** Salivary biomarkers in periodontitis.

Salivary biomarker	Function	References
IL-1	*Strong relation with periodontal disease; high inflammatory potential*	[25, 28–33, 45]
IL-6	*Stimulates osteoclastic differentiation; increased levels in periodontal disease; regulated the immune responses*	[34–38, 45]
Tumor necrosis factor	*Influences the bone collagen synthesis*	[28, 39, 45]
Matrix metalloproteinase-9	*Mediator of the immune response and tissue destruction in periodontal disease*	[40–43]

**Table 4:** Salivary biomarkers in oral cancer.

Salivary biomarker	Variation	References
IL-8	*High levels*	[53–56]
Endothelin receptor type-B hypermethylation	*High levels*	[52]
microRNAs (miR-200a, miR-125a, and miR-31)	*High levels*	[53, 54]
S100 calcium-binding protein P	*High levels*	[56]
IL-1beta	*High levels*	[56]
Tissue polypeptide antigen (TPA)	*High levels*	[56]
Cancer antigen CA125	*High levels*	[57, 59]
p53 antibodies	*High levels*	[58]
H3 histone family 3A	*High levels*	[56]
Cyfra 21-1	*High levels*	[57]
Ornithine decarboxylase antizyme 1	*High levels*	[56]
